# Comparison of co-administration of amiodarone and rivaroxaban to co-administration of dronedarone and rivaroxaban for hemorrhage risks after atrial fibrillation ablation

**DOI:** 10.1007/s10840-022-01128-w

**Published:** 2022-01-20

**Authors:** Peng Zhang, Maojing Wang, Wenheng Liu, Pin Sun, Shanglang Cai, Yaqi Pan, Qing Zhao

**Affiliations:** 1 Department of Cardiology, The Affiliated Hospital of Qingdao University, Qingdao , 266000 Shandong China; 2 Department of Cardiac Ultrasound, The Affiliated Hospital of Qingdao University, Qingdao, 266000 Shandong China

**Keywords:** Atrial fibrillation, Dronedarone, Amiodarone, Rivaroxaban, Hemorrhage

## Abstract

**Purpose:**

To investigate whether co-administration of antiarrhythmic dronedarone and anticoagulant rivaroxaban would increase the risks of hemorrhage after atrial fibrillation (AF) ablation.

**Methods:**

A total of 100 patients with AF who underwent radiofrequency catheter ablation (CA) in the Department of Cardiology, the Affiliated Hospital of Qingdao University from 2019–12 to 2020–11 were included. Patients were divided into an oral dronedarone and rivaroxaban group (D-R group, *N* = 50) and an oral amiodarone and rivaroxaban group (A-R group, *N* = 50) according to the postoperative antiarrhythmic and anticoagulation strategies. Patients in 2 groups were given propensity score matching (PSM) to obtain a sample with balanced inter-group covariates. A retrospective observational study was conducted. After 3 months of follow-up, the incidence of clinically relevant non-major bleeding (CRNMB), major hemorrhages, and early AF recurrence was observed.

**Results:**

After PSM, 41 patients were included in each group. With similarly distributed baseline characteristics and ablation characteristics after PSM, the CRNMB rate after AF ablation was significantly higher in the D-R group than in the A-R group (26.8% versus 7.3%, *P* = 0.02), and no major hemorrhages were detected in both groups. No significant difference was observed in the sinus rhythm maintenance rate between the D-R group and the A-R group (26.8% vs. 22.0%, *P* = 0.43).

**Conclusions:**

Compared to co-administration of amiodarone and rivaroxaban, co-administration of dronedarone and rivaroxaban increases the risk of CRNMB but it does not increase the risk of major hemorrhages in blanking period after AF ablation.

## Introduction

Catheter ablation (CA) is a well-established treatment for the prevention of atrial fibrillation (AF) recurrences [1]. Dronedarone, an oral class III antiarrhythmic drug without iodine atoms in its structure, is often used to maintain sinus rhythm after CA and does not increase the incidence of thyroid toxicity [2]. However, dronedarone increases the plasma levels of direct oral anticoagulants (DOACs), such as rivaroxaban [3]. There are limited data on hemorrhage complications after co-administration of dronedarone and rivaroxaban. The current observational study evaluated whether co-administration of dronedarone and rivaroxaban would increase hemorrhage risks compared to co-administration of amiodarone and rivaroxaban after CA.

## Methods

### Patients

A total of 100 patients who had indication for CA and underwent CA for the first time in the Affiliated Hospital of Qingdao University from December 2019 to November 2020 were retrospectively reviewed. The patients were divided into an oral dronedarone and rivaroxaban group (D-R group, *N* = 50), and an oral amiodarone and rivaroxaban group (A-R group, *N* = 50) according to the postoperative antiarrhythmic and anticoagulation strategies.

The inclusion criteria were as follows: (1) patients with paroxysmal AF or persistent AF; (2) patients with nonvalvular AF. The exclusion criteria were as follows: (1) left atrial thrombosis detected by transesophageal echocardiography; (2) valvular AF (AF with moderate to severe mitral stenosis or mechanical valve replacement); (3) permanent AF; (4) warfarin use during the perioperative period; (5) major bleeding complications during surgery; (6) acute heart failure; (7) severe thyroid, lung, liver, and/or kidney dysfunction; and (8) age < 18 or > 75 years.

Patients in both groups were given propensity score matching (PSM) to obtain a sample with balanced inter-group covariates. The matching factors included age, AF types, left atrial diameter, left ventricular ejection fraction, cerebral embolism, hypertension, diabetes mellitus, CHA_2_DS_2_-VASc score, HAS-BLED score, preoperative medication, ablation characteristics, and procedural complications. According to the propensity score, the D-R group and the A-R group were matched at a ratio of 1:1. The matching tolerance was set to 0.02.

### Radiofrequency catheter ablation methods

All patients underwent transesophageal echocardiography within 48 h before ablation to exclude left atrial thrombosis. The right femoral vein was punctured, a 10-electrode coronary sinus catheter was placed in the coronary sinus, and then a Swartz 8.5 F sheath was inserted into the right atrium. The atrial septum puncture needle was guided along the sheath to the oval fossa to puncture the atrial septum. After a successful puncture, 100 IU/kg unfractionated heparin sodium was injected intravenously. The activated whole blood coagulation time (ACT) was monitored once every 0.5 h during the procedure. The dosage of unfractionated heparin sodium and protamine needed during the procedure was adjusted to stabilize the ACT value at 250 ~ 350 s. The Swartz sheath was placed in the left atrium, and star mapping electrodes were placed along the sheath in the left atrium. The left atrium model was established under the guidance of the Carto system.

The ablation strategy of paroxysmal AF was pulmonary vein isolation (PVI). A 3.5-mm ablation catheter (Smart Touch Johnson & Johnson, New Brunswick, NJ, USA) was used to perform PVI in the bilateral pulmonary veins. The discharge energy in the anterior wall was 40 W, the pump tube flow rate was 20 mL/min, and the limited ablation temperature was 43 ℃; consequently, the ablation was stopped when the ablation index (AI) reached 450 ~ 550. Furthermore, when the discharge energy was 35 W at the top and posterior walls, the pump tube flow rate was 17 mL/min, and the limited ablation temperature was 43 ℃; ablation was stopped when AI reached 350 ~ 400. Ablation was successful when both pulmonary veins reached bidirectional electrical isolation. Patients who did not convert to sinus rhythm successfully after PVI were subjected to direct current synchronized cardioversion (DCC).

The ablation strategy of persistent AF was PVI + left atrial apical line and mitral isthmus line ablation. The PVI ablation method was the same as for paroxysmal AF. When the parietal line of the left atrium was ablated and the ablation energy was 30 W, the pump tube flow rate was 17 mL/min, the limited ablation temperature was 43 ℃, and the ablation was stopped when AI reached 400; when the ablation energy was 35 W, the pump tube flow rate was 17 mL/min, the limited ablation temperature was 43 ℃, and the ablation was stopped when AI reached 400 ~ 450. Ablation was successful when both sides of the ablation line reached bidirectional electrical isolation, following which the patients with AF were given DCC after ablation.

### Perioperative antiarrhythmia and anticoagulation methods

All patients were administered rivaroxaban 15 mg orally for at least 1 month before CA, which was stopped 1 day before the procedure, and the administration of antiarrhythmic drugs was stopped at five half-lives before the procedure. Under the condition of no bleeding complications, patients with a history of thyroid dysfunction, including hypothyroidism, subclinical hypothyroidism, and hyperthyroidism, were administered dronedarone and rivaroxaban orally 6 h after the procedure. For these patients, 400 mg dronedarone was given twice a day, and 15 mg rivaroxaban was given once a day. Patients with normal thyroid dysfunction were administered amiodarone and rivaroxaban orally 6 h after the procedure. The dosage of amiodarone was 200 mg three times a day for the first 10 days and then it was adjusted to 200 mg once a day after the first 10 days, and 15 mg rivaroxaban was taken once a day. If the patient had bleeding complications during the operation, only dronedarone or amiodarone was administered after the procedure. The thromboembolism risks and bleeding risks were evaluated according to CHA_2_DS_2_-VAS score and HAS-BLED score. The maximum delay for oral anticoagulants was 48 h if the risks of bleeding outweigh the risks of thromboembolism especially in patients with HAS-BLED score ≥ 3. All patients took antiarrhythmic drugs and anticoagulants orally for at least 3 months after CA.

### Postoperative follow-up

All patients returned for follow-up at the outpatient clinic for 3 months after ablation. The main follow-up data collected included symptoms, signs, and tests of hemorrhage. Monthly body surface electrocardiogram and 24-h Holter monitoring were performed to record the recurrence of AF. Patients were instructed to immediately obtain a surface electrocardiogram at a local hospital if symptoms such as palpitation, chest tightness, or feelings of suffocation were suspected to be an AF recurrence.

### Data collection

**Fig. 1:**
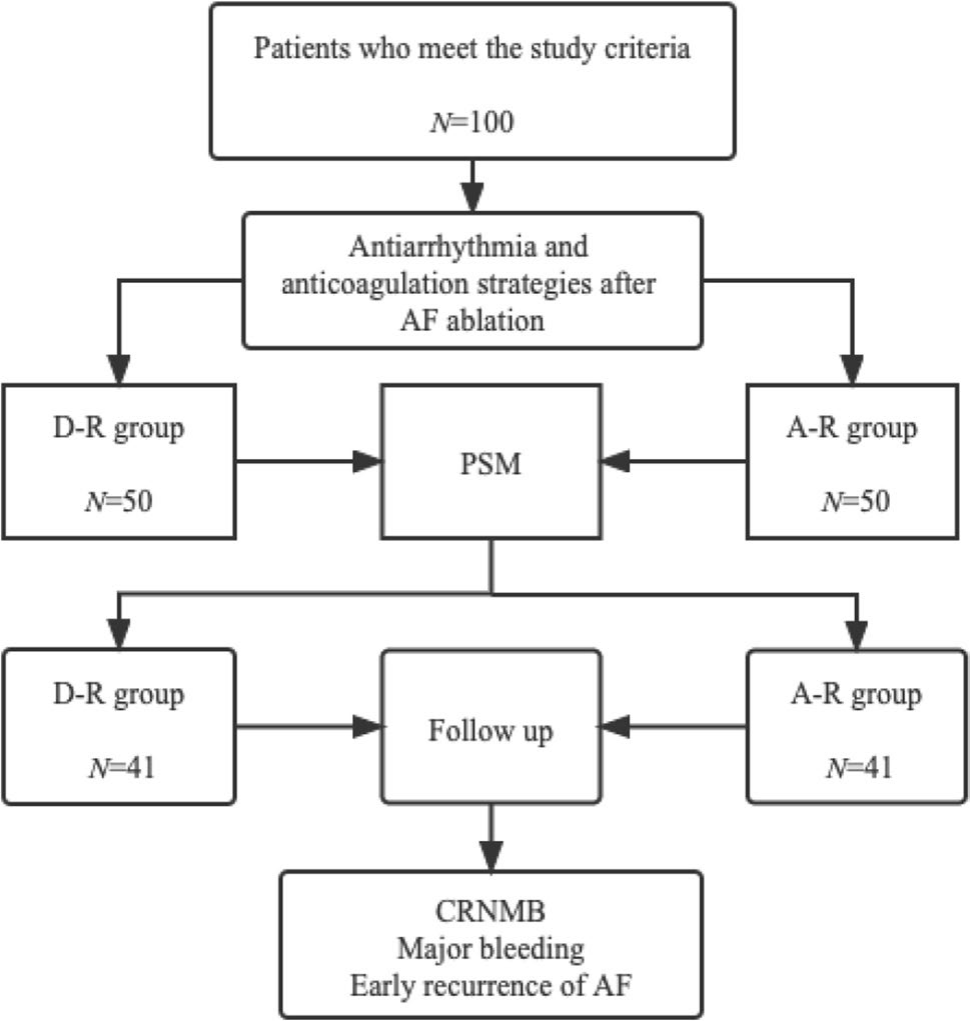
Strategy flow chart for comparison of A-R group and D-R group. AF, atrial fibrillation; PSM, propensity score matching

The observation indicators included as follows: (1) Clinically relevant non-major bleeding (CRNMB) was defined according to the criteria of Bleeding Academic Research Consortium (BARC) as any overt, actionable sign of hemorrhage that does not fit the criteria for types 3, 4, or 5 but meet at least one of the following criteria: (a) requiring nonsurgical, medical intervention by a healthcare professional, (b) leading to hospitalization or increased level of care, (c) prompting evaluation [4]. For example, petechia (subcutaneous bleeding < 2 mm in diameter), purpura (subcutaneous bleeding with a diameter of 3 ~ 5 mm), ecchymosis (subcutaneous bleeding over 5 mm in diameter), small subcutaneous hematoma (hematoma diameter ≥ 3 cm measured by ultrasound), minor gastrointestinal hemorrhage (only positive by a fecal occult blood test, no melena or hematemesis), epistaxis, and other bleeding events that do not require blood transfusion or surgery and do not increase the number of hospitalization days; (2) Major bleeding was defined according to the criteria of International Society on Thrombosis and Haemostasis (ISTH) and BARC as clinically overt bleeding which was fatal or associated with any of the following: (a) a fall in hemoglobin level of 2 g/dL or more or documented transfusion of at least 2 units of packed red blood cells, (b) involvement of a critical anatomical site (intracranial, spinal, ocular, pericardial, articular, intramuscular with compartment syndrome, retroperitoneal), (c) BARC types 3 to 5 were also considered a major bleeding [5]; (3) Early recurrence of AF: The first 3 months after the first ablation was a blank period during which any atrial arrhythmia (AF, atrial flutter, and atrial shock) occurrence was counted as early recurrence of AF. A strategy flow chart is illustrated in Fig. 1.

### Statistical analysis

The statistical data of this study were processed using SPSS 19.0 and Prism 8.0 statistical software. The continuous variables are expressed as the mean ± standard deviation and were compared using single-factor *t*-test statistics. Categorical variables are expressed as numbers and percentages and were processed using the *χ*^2^ test. Kaplan–Meier curves were used to display the cumulative bleeding complication-free survival and cumulative atrial arrhythmia-free survival after the first ablation. *P* < 0.05 indicates a statistically significant difference.

## Results

### Patient characteristics

After PSM, 41 patients were included in each group. As shown in Table 1, the distributions of the patient baseline data did not show any statistically significant difference between the groups after PSM.

**Table 1:** Comparison of baseline clinical characteristics

	Before PSM			After PSM		
	A-R group (*N* = 50)	D-R group (*N* = 50)	*P*	A-R group (*N* = 41)	D-R group (*N* = 41)	*P*
Age (years)	61.32 ± 7.15	62.92 ± 8.10	0.30	61.42 ± 6.74	61.93 ± 8.11	0.71
Men	25 (50.0%)	22 (44.0%)	0.55	18 (43.9%)	18 (43.9%)	-
Paroxysmal atrial fibrillation	29 (58.0%)	32 (64.0%)	0.54	30 (73.2%)	25 (61.0%)	0.24
Left atrial diameter (mm)	40.20 ± 3.49	40.02 ± 3.40	0.79	39.90 ± 3.53	39.95 ± 3.60	0.95
Left ventricular ejection fraction (%)	60.26 ± 4.20	59.92 ± 3.13	0.65	59.63 ± 3.15	59.76 ± 2.36	0.84
Cerebral embolism	3 (6.0%)	4 (8.0%)	0.70	2 (5.0%)	2 (5.0%)	-
Hypertension	24 (48.0%)	20 (40.0%)	0.42	20 (48.8%)	16 (39.0%)	0.37
Diabetes	20 (40.0%)	15 (30.0%)	0.30	14 (34.1%)	13 (31.7%)	0.81
CHA_2_DS_2_-VASc score	1.90 ± 1.04	2.06 ± 1.20	0.48	1.93 ± 1.08	1.88 ± 1.10	0.84
HAS-BLED score	1.20 ± 1.11	1.18 ± 0.98	0.92	1.07 ± 0.93	1.15 ± 1.04	0.74
Preoperative medication						
Propafenone	6 (12.0%)	3 (6.0%)	0.30	6 (14.6%)	3 (7.3%)	0.29
Beta blocker	13 (26.0%)	11 (22.0%)	0.64	12 (29.3%)	9 (22.0%)	0.45
Dronedarone	4 (8.0%)	6 (12.0%)	0.51	2 (5.0%)	6 (14.6%)	0.14
Amiodarone	7 (14.0%)	9 (18.0%)	0.59	6 (14.6%)	6 (14.6%)	-
Calcium channel blocker	2 (4.0%)	5 (10.0%)	0.24	2 (5.0%)	4 (9.8%)	0.40

Data are presented as mean + standard deviation or *n* (%)

All patients successfully converted to sinus rhythm after CA. No cerebral embolism, pulmonary embolism, transient ischemic attack, cardiac tamponade, or major bleeding complications developed during the operation after PSM. There was no difference between D-R group and A-R group in minor bleeding complications, including small puncture site bleeding, hematoma, or ecchymosis (Table 2). All the patients who suffered from minor bleeding complications completely recovered within 2 days after surgery.

**Table 2:** Comparison of ablation characteristics and procedural complications

	Before PSM			After PSM		
	A-R group (*N* = 50)	D-R group (*N* = 50)	*P*	A-R group (*N* = 41)	D-R group (*N* = 41)	*P*
Operation time (min)	122.76 ± 18.02	124.32 ± 17.10	0.71	122.49 ± 14.82	123.98 ± 15.14	0.65
Ablation time (min)	35.44 ± 5.16	35.56 ± 6.70	0.94	34.83 ± 4.31	34.80 ± 5.83	0.98
X-ray exposure time (min)	4.76 ± 0.87	4.59 ± 0.71	0.43	4.52 ± 0.81	4.29 ± 0.77	0.19
Direct current synchronized cardioversion	12 (24.0%)	11 (22.0%)	0.81	10 (24.4%)	11 (26.8%)	0.80
Success rate of instant pulmonary vein isolation	100%	100%	-	100%	100%	-
Cerebral embolism	0	1	0.32	0	0	-
Pulmonary embolism	0	0	-	0	0	-
Transient ischemic attack	0	0	-	0	0	-
Cardiac tamponade	0	0	-	0	0	-
Minor bleeding complications	6 (12.0%)	8 (16.0%)	0.56	6 (14.6%)	6 (14.6%)	-
Major bleeding complications	0	0	-	0	0	-

Data are presented as mean + standard deviation or *n* (%)

### Outcomes

**Fig. 2:**
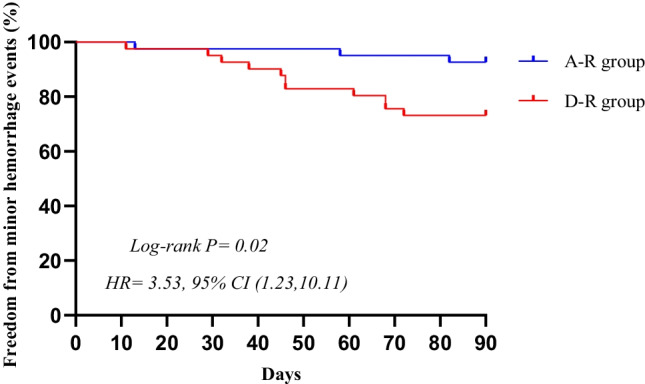
Freedom from minor hemorrhage after AF ablation for D-R group and A-R group. At 3-month follow-up, there were differences between the two groups (*P* = 0.02). AF, atrial fibrillation

During 3-month of follow-up, D-R group had a significantly higher CRNMB rate than A-R group (26.8% versus 7.3%, *P* = 0.02). Minor hemorrhage events occurred in 11 patients in D-R group, including petechia in 3 cases, purpura in 4 cases, and ecchymosis in 2 cases. Minor gastrointestinal hemorrhage occurred in 1 case, and epistaxis occurred in 1 case. Three cases of minor hemorrhage events were noted in A-R group, including petechia in 1 case, purpura in 2 cases, and a small subcutaneous hematoma in 1 case. However, the rates of petechia, purpura, ecchymosis, small subcutaneous hematoma, minor gastrointestinal hemorrhage, and epistaxis were numerically but not significantly different between the two groups. The minor hemorrhage-free survival rates of the two groups are shown in Fig. 2. No major bleeding complications developed in either group during follow-up. Details of the hemorrhage events are presented in Table 3.

**Table 3:** Comparison of hemorrhage events during follow-up

	A-R group (*N* = 41)	D-R group (*N* = 41)	*P*
Minor hemorrhage events	3 (7.3%)	11 (26.8%)	0.02
Petechia	1 (2.4%)	3 (7.3%)	0.31
Purpura	1 (2.4%)	4 (9.8%)	0.24
Ecchymosis	0	2 (4.9%)	0.56
Small subcutaneous hematoma	1 (2.4%)	0	0.32
Minor gastrointestinal hemorrhage	0	1 (2.4%)	0.32
Epistaxis	0	1 (2.4%)	0.32
Major bleeding events	0	0	-

Data are presented as *n* (%)

**Fig. 3:**
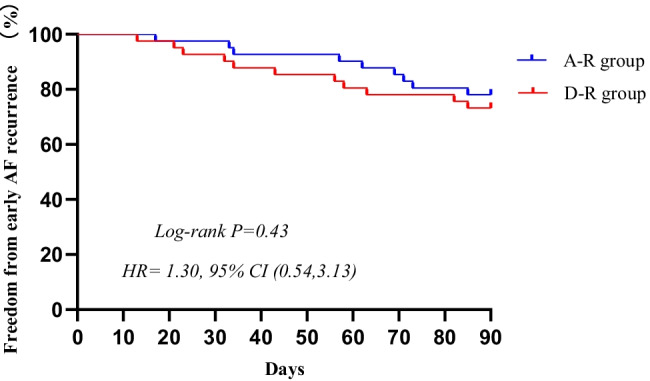
Freedom from early AF recurrence after AF ablation for D-R group and A-R group. At 3-month follow-up, there were no differences between the two groups (*P* = 0.43). AF, atrial fibrillation

During 3-month of follow-up, the early AF recurrence rate was numerically but not significantly higher in the D-R group than in the A-R group (26.8% vs. 22.0%, *P* = 0.43). The early AF recurrence-free survival rates of the two groups are shown in Fig. 3.

## Discussion

The main findings of the present observational, single-center study are as follows: (1) Co-administration of dronedarone and rivaroxaban yielded a significantly higher risk of CRNMB for patients with paroxysmal or persistent AF but did not increase the risk of major hemorrhage after CA. (2) Dronedarone did not increase the early AF recurrence rate after the first ablation compared to amiodarone.

AF catheter ablation is effective in maintaining sinus rhythm in patients with paroxysmal and persistent AF [6, 7]. The first 3 months after the first ablation was a blanking period during which any atrial arrhythmia occurrence was not counted as an AF recurrence but was the strongest predictor for AF recurrence in the future [8]. Continuing antiarrhythmic drug treatment for 3 months after CA was recommended to reduce early AF recurrences during the blanking period [9]. Additionally, oral anticoagulant therapy is continued for at least 2 months following CA in all patients [10].

The plasma levels of DOACs can be influenced by various drug-drug interactions and consequently influence anticoagulant activity [11]. At present, the interactions between amiodarone and DOACs have been noted [12]. However, relevant data on co-administration of dronedarone and rivaroxaban are lacking. The present study demonstrated that co-administration of dronedarone and rivaroxaban increased the CRNMB risks, but it is still a relatively safe treatment option because it did not increase the incidence of major bleeding events in short-term applications. In a recent study of 23 patients with paroxysmal AF for an average 9.1-month follow-up, concomitant use of dronedarone and rivaroxaban was not associated with significant adverse events, including major bleeding [13]. A multicenter study found that co-administration of dronedarone and DOACs does not increase the risk of massive hemorrhage [14], which is consistent with our results. An increase in the blood drug concentration of DOACs might not be related to an increased risk of massive hemorrhage.

Rivaroxaban is mainly resecreted into the gut via a P-glycoprotein transporter and metabolized by cytochrome P3A4 (CYP3A4) [15]. Dronedarone is an inhibitor of P-glycoprotein and CYP3A4 [12], which may result in higher plasma levels of rivaroxaban and lead to an increased risk of CRNMB. Regardless, co-administration of dronedarone and rivaroxaban can be used as a rhythm-controlling and anticoagulation strategy after CA, but the signs and tests of bleeding need to be monitored in real time.

Amiodarone is the most effective antiarrhythmic drug and shows lower AF recurrence than dronedarone [16], but numerous extracardiac side effects, such as thyroid toxicity, make it a second-line treatment [17]. Although less effective than amiodarone in rhythm control, dronedarone may be a preferable first choice with few extracardiac side effects [18]. The present study showed that dronedarone and amiodarone have comparable abilities to maintain sinus rhythm in the blanking period after CA. However, it is currently thought that dronedarone is less efficacious than amiodarone in maintaining sinus rhythm [19]. A possible explanation for the difference in results is the short follow-up time in this study; only the recurrence rate of atrial fibrillation in blanking period was evaluated. The recurrence of AF in blanking period cannot be used as an indicator to evaluate the effect of antiarrhythmic drugs.

The present study is limited by its single-center nature. A larger multicenter trial should be performed to elevate the hemorrhage risks of co-administration of dronedarone and rivaroxaban. Moreover, the judgment of AF recurrence mainly depends on symptoms and 24-h Holter monitoring, which does not rule out the possibility that patients with mild symptoms are not undergoing electrocardiogram examinations when AF recurs.

## Conclusions

Compared to co-administration of amiodarone and rivaroxaban, co-administration of dronedarone and rivaroxaban increases the risk of CRNMB but it does not increase the risk of major hemorrhage in blanking period after CA.

## Abbreviations

AF: Atrial fibrillation
DOACs: Direct oral anticoagulants
ACT: Activated whole blood coagulation time
CA: Catheter ablation
PVI: Pulmonary vein isolation
AI: Ablation index
DCC: Direct current synchronized cardioversion
CRNMB: Clinically relevant non-major bleeding
BARC: Bleeding Academic Research Consortium
ISTH: International Society on Thrombosis and Haemostasis
PSM: Propensity score matching

## Declarations

### Data access statement

All data are provided in full in the results section of this paper.

This study complied with the 1975 Helsinki Ethics Guide and was approved by the Ethics Committee of The Affiliated Hospital of Qingdao University (No. QYFYWZLL 26013). Informed consent was obtained from the patients and their families.
